# On the Optical Activity of Poly(l-lactic acid) (PLLA) Oligomers and Polymer: Detection of Multiple Cotton Effect on Thin PLLA Solid Film Loaded with Two Dyes

**DOI:** 10.3390/ijms22010008

**Published:** 2020-12-22

**Authors:** Franco Cataldo

**Affiliations:** Actinium Chemical Research Institute, Via Casilina 1626a, 00133 Rome, Italy; franco.cataldo@fastwebnet.it

**Keywords:** poly(l-lactic acid) (PLLA), optical rotatory dispersion (ORD), polycondensation, racemization, thin solid film, exciton coupling, organic electronics

## Abstract

Optical rotatory dispersion (ORD) is a beautiful analytical technique for the study of chiral molecules and polymers. In this study, ORD was applied successfully to follow the degree of polycondensation of *l*-(+)-lactic acid toward the formation of poly(lactic acid) oligomers (PLAO) and high molecular weight poly(l-lactic acid) (PLLA) in a simple esterification reaction equipment. PLLA is a biodegradable polymer obtainable from renewable raw materials. The racemization of the intrinsically isotactic PLLA through thermal treatment can be easily followed through the use of ORD spectroscopy. Organic or molecular electronics is a hot topic dealing with the combination of π-conjugated organic compounds and polymers with specific properties (e.g., chirality) which can be exploited to construct optoelectronic devices, such as organic light-emitting diodes (OLEDs), organic photovoltaic (OPV) high efficiency cells, switchable chirality devices, organic field-effect transistors (OFETs), and so on. ORD spectroscopy was applied to study either the gigantic optical rotation of PLLA films, as well as to detect successfully the excitonic coupling, occurring in thin solid PLLA green film loaded with a combination of two dyes: SY96 (a pyrazolone dye) and PB16 (the metal-free phthalocyanine pigment). The latter compound PLLA loaded with SY96 and PB16 shows a really gigantic optical activity in addition to typical ORD signal due to exciton coupling and may be considered as a simple and easily accessible model composite of a chiral polymer matrix combined with π-conjugated dyes for molecular electronics studies.

## 1. Introduction

Poly(l-lactic acid) (PLLA) or poly(lactide) (PLA) is a biodegradable polymer synthesized from raw materials from renewable sources [1,2,3,4,5,6,7,8,9,10,11]. Optically active *l*-(+)-lactic acid is produced by fermentation with suitable lactobacillus and a number of different biomass substrates [12]. More in detail, suitable biomasses for lactic acid production span from hexoses in general, glucose *in primis*, followed by molasses, sugar beet juice, sulfite liquors, and even whey [12]. Of course, also starches from potato, wheat, and rice are other suitable substrates [12]. Homolactic fermentation (one molecule of glucose yields two molecules of lactic acid) generally leads to *l*-(+)-lactic acid, while heterolactic fermentation (one molecule of glucose yields one molecule of lactic acid and other products, e.g., CO_2_ and ethanol) permits the access to the other enantiomer *d*-(−)-lactic acid [12]. Thus, also the access to poly(d-lactic acid) (PDLA), in addition to the most common PLLA, is ensured. In recent years, great progresses were reached also from blending or copolymerizing PLLA with PDLA in terms of mechanical and thermal properties modification and improvements [9]. Of course, racemic lactic acid can also be produced through industrial chemical processes starting from acetaldehyde, propylene, or propionic acid [12], ensuring the access to amorphous and atactic PLA. Perhaps, the most comprehensive and updated review on PLLA and related compounds is represented by the two Springer’s book volumes published in 2018 [10,11]. The industrial production of PLLA had already started in the nineties [1], initially for niche applications, mainly in the medical field (like suture threads), and it has now exploded in many other application fields, especially in packaging and related fields [2,3,4,5,6,7,8], production of cardiovascular devices, tissue engineering, including skeletal tissue, controlled drug delivery, 3D printing, and automotive applications [11]. PLLA conjugates, in a single material, excellent mechanical properties [13] with very good thermal properties [14,15] with efficient biodegradability [4].

Poly(l-lactic acid) (PLLA) is a stereoregular polymer intrinsically isotactic due to the spatial structure of the monomer l-lactic acid. It was found in a conclusive way, through vibrational circular dichroism (VCD), that PLLA assumes helical conformation in chlorinated solvents [16,17], as well as in certain polar solvents, like dimethylsulfoxide [16,17], confirming previous preliminary results obtained with optical rotatory dispersion (ORD) spectroscopy [18]. The macromolecular dynamics of PDLA and PLLA in dilute solutions was also studied by intrinsic viscosity, small angle X-ray scattering and static light scattering in a medium polar solvent like tetrahydrofuran and fine details of helical conformation were revealed [19,20]. Thus, PLLA and PDLA are ideal polymers for the study of the extrinsic Cotton effect or induced Cotton effect involving the interaction of helical polymer structure in solution with a guest molecular chromophore hosted inside the spiral pitches or intercalated between the PLLA helices. The guest symmetric molecule without ORD signal, by interacting with the asymmetric helical structure of PLLA, gives rise to an induced ORD signal in correspondence of its electronic transition in the visible through the coupled oscillator mechanism or the one-electron mechanism or exciton coupling [21,22,23,24,25]. In other words, the electronic transition of the chromophore of the guest molecule has a spatial relationship with host macromolecule which is manifested as a dissymmetry in the ORD spectrum of the complex [21,22,23,24,25]. The induced Cotton effect was indeed detected by ORD spectroscopy both in solutions of PLLA/iodine [26] and PLLA/C_60_ fullerene [27], whereas a charge-transfer host-guest interaction was measured in both cases.

In the present work, the optical activity or PLLA is covered from different points of view. First of all, it will be shown that the oligomerization progress of *l*-(+)-lactic acid can be followed by ORD spectroscopy in solution till the polymerization to a high molecular weight PLLA polymer. In contrast, the early work on ORD properties of PLLA [18] was focused exclusively to high molecular weight PLLA solution. The lactic acid oligomers (PLAO) have interesting potential applications as plasticizers of PLLA [28,29] and in improving its barrier properties in packaging applications [30]. The thermal stability of PLAO was subjected to a thorough investigation [14]. Then, the PLLA optical activity was studied in thin solid films derived from recycled PLLA water bottles, showing not only the anomalous and multiple Cotton effect due to a pigment embedded in the PLLA matrix but also gigantic solid state ORD properties which are easily accessible and may be exploited in new chirooptical devices involving display and wearable technologies, as well as the promise of efficient, large-area solar cells [31].

## 2. Results and Discussion

### 2.1. Optical Rotatory Dispersion Measurement on PLAO during Lactic Acid Polycondensation

**Figure 1:**
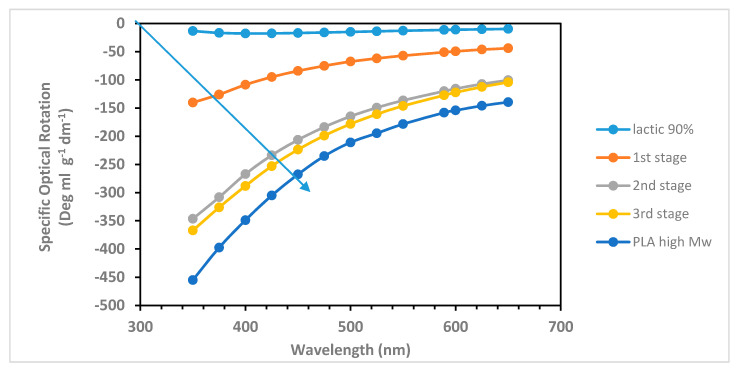
Optical rotatory dispersion (ORD) of poly(lactic) acid oligomers (PLAO) at different degrees of oligomerization. The arrow shows the growth in optical activity as function of the molecular weight increase, starting from the ORD curve of 90% *l*-(+)-lactic acid (neat, light blue dots at the top of the figure) to a high molecular weight poly(l-lactic acid) (PLLA) dissolved in CHCl_3_ (1.50 g/100 mL; dark blue dots at the bottom of the figure). The ORD curves of the PLAO oligomers were recorded in acetone solvent, with the exception of the last curve in yellow dots, which required the use of CHCl_3_ solvent (in all cases, 1.50 g in 100 mL).

Commercial lactic acid 88–92% was subjected to a series of azeotropic distillation, batchwise, without the addition of any catalyst. Initially, toluene was used as water entrainment agent in the first stage, while, in the following stages, xylenes were employed, which have higher boiling point than toluene and ensure a higher temperature for the polycondensation reaction. It is well known that commercial lactic acid 90% has a residual water content of 17% by weight [32], but it also contains lactoyllactic acid (the linear dimer of lactic acid), as well as trimers and tetramers of lactic acid [12,32]. Instead, the cyclic dimers of lactic acid, the dilactides, are produced only by heating under reduced pressure and in presence of a catalyst [12,32]. In Figure 1, the ORD curve of 90% lactic acid solution (light blue dots at the top of the figure) appears as a flat curve with a relatively weak specific optical rotation in all the wavelengths range considered. It is worth the reminder, here, that the specific optical rotation at any wavelength [α]_λ_ is defined as follows:

[α]_λ_ = α l^−1^ c^−1^, (1)

where α is the rotation degree of the plane of polarized light crossing a cell (containing the sample solution with a concentration c expressed in g/mL) having a length l expressed in dm [33]. The subscript λ refers to the selected wavelength used to measure the specific optical rotation; in the present case, λ is comprised between 350 and 650 nm.

After the first azeotropic distillation stage (Figure 1, orange curve), the specific optical rotation of the ORD curve is increased dramatically, especially at shorter wavelengths. Furthermore, the water removed exceeds the amount of free water present in 90% lactic acid, and it is possible to calculate that at least 13% of the lactic acid groups are already esterified. This implies that, at this stage, not only 100% nominal lactic acid concentration has been reached, but already a mixture of linear oligomers is present in the product. After all, we have measured [α]_589_ = −11.52 in 90% lactic acid solution and after the first polycondensation step [α]_589_ = −51.1 (see Figure 1). It is known that l-lactide is characterized by a large specific optical rotation, i.e., [α]_589_ = −266.3 in CH_2_Cl_2_ [34], but, considering the mild reaction conditions adopted, it is reasonable to affirm that the presence and contribution of the l-lactide in the reaction mixture is negligible. Instead, it is interesting to mention the work Kimura et al. [35], which has shown that the helical structure appears already in oligo(l-lactic acid) octamers. In the second polycondensation step, this time performed with xylenes as water entrainment agent, on the basis of the recovered reaction water, it can be calculated that 60% of the starting lactic acid has been oligomerized. Indeed, Figure 1 (grey curve) shows further deep enhancement in the optical activity with a large shift of the [α]_λ_ toward negative values in the ORD curve and, for example, [α]_589_ = −120.1.

In the third polycondensation stage, the water removal became more difficult, and the degree of lactic acid autoesterification was calculated at about 70%. Figure 1 (yellow curve) shows the ORD curve further shifted toward lower [α]_λ_ values, for example, by considering the specific optical rotation commonly read at 589 nm: [α]_589_ = −127.4. This suggests that the PLAO chains are now considerably long to assume larger helical structures with respect to the previous shorter chain oligomers. At this stage, all the optical activity observed is due to the helical conformation of the PLAO since the contribution of the monomer *l*-(+)-lactic acid optical activity is negligible. Figure 1 shows also the ORD curve of a commercial high molecular weight PLLA (dark blue curve) with 100% esterified lactic acid functionalities. In this latter case [α]_589_ = −158.2, reading the specific optical rotation of PLLA at the D-line of sodium, the classic wavelength used in polarimetry. As already stated in the introduction, PLAO have potential as a plasticizer of PLLA [28,29] and probably in other polymer matrices. Furthermore, PLAO seems effective in improving PLLA barrier properties in packaging applications [30]. In this applicative perspective, ORD on PLAO could be a powerful and effective tool to control the degree of oligomerization and even the full polymerization of PLLA.

### 2.2. The Progress of Lactic Acid Polycondensation Can Be Followed Also by FT-IR Spectroscopy

**Figure 2:**
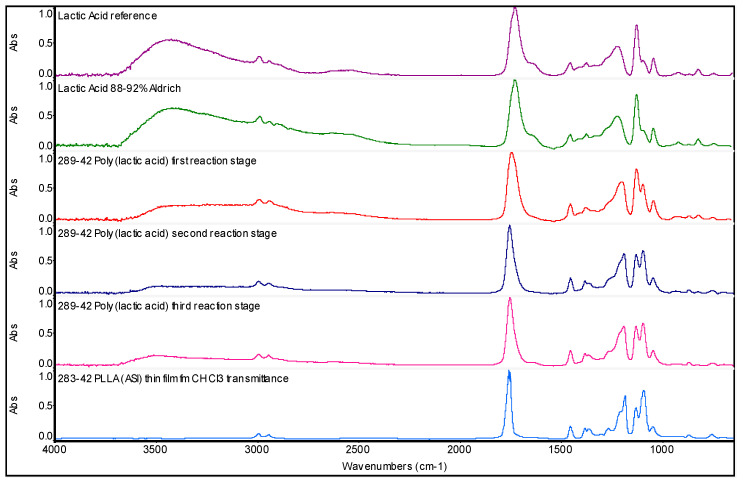
FT-IR spectra (from top to bottom): Lactic acid 100% (violet); Lactic acid 90% (green); PLAO first reaction stage (red); PLAO second reaction stage (dark blue); PLAO third reaction stage (amaranth); PLLA (ASI) high molecular weight reference polymer (light blue).

The progress of lactic acid polycondensation reaction can be followed also by infrared spectroscopy in addition to ORD spectroscopy. As shown in Figure 2, the FT-IR spectra (from top to bottom) show a gradual decrease of the intensity of the hydroxyl stretching band at about 3450 cm^−1^ with the progress of the polycondensation reaction. This is logical since less OH groups are present in the reaction mixture by increasing the degree of polymerization. Indeed, the OH groups are almost no more detectable in the case of the high molecular weight PLLA.

The integration of the band at 3450 cm^−1^ permits to follow, in a quite straightforward way, the progress of the polymerization from lactic acid to PLAOs to PLLA, as shown in Table 1. Lactic acid shows, in Figure 2, a broad band at about 1750 cm^−1^ due to the ketone stretching mode of the carboxylic acid group [36]. However, with the progress of the polymerization, the mentioned infrared absorption band became narrower. Thus, the integration of this band (see Table 1) gives a very nice trend toward the high molecular weight PLLA, which is characterized exclusively by the presence of ester groups and the virtual absence of the free carboxylic functionality.

The infrared band at 1129 cm^−1^ is due to the C-OH bending mode. In Table 1, it is possible to follow the trend of the area of this infrared band as function of the progress of the polycondensation. As in the case of the OH stretching band, also, this absorption band becomes smaller since the free OH groups are consumed in the polycondensation reaction. In Table 1 are also reported three infrared absorption bands related to the ester group [36] and located at 1456, 1224, and 1096 cm^−1^, in which integrated absorptivity grows with the increase of the molecular weight of PLAOs till the final stage of PLLA.

### 2.3. Partial PLLA Racemization by Thermal Treatment in Selected Solvent Followed by ORD Spectroscopy

**Figure 3:**
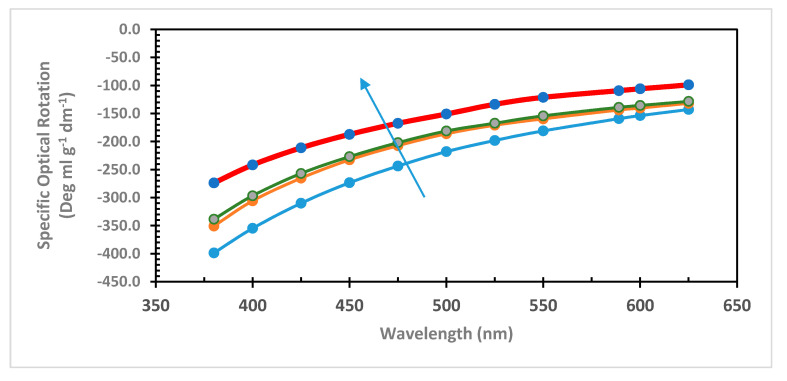
Optical rotatory dispersion (ORD) of pristine PLLA in CHCl_3_ (blue line and dots); PLLA heated 8 h in chlorobenzene (orange line and dots); PLLA heated 4 h in o-dichlorobenzene (green line and grey dots); PLLA heated 28 h in 1,2,4-trichlorobenzene (red line and blue dots). The arrow over the ORD curves indicates the PLLA racemization trend.

The racemization of PLLA was studied under thermal treatment [37,38] or presence of catalysts [37]. Racemization of PLLA was also detected during photodegradation [39], aqueous hydrolysis [40], and during depolymerization [41]. In our attempt to cause the partial racemization of PLLA, we selected three different solvents, monochorobenzene (ClBz, boiling point 132 °C), o-dichlorobenzene (o-DCBz, boiling point 180 °C), and 1,2,4-trichlorobenzene (1,2,4-TCBz, boiling point 214 °C). In a purely explorative work, PLLA was heated in these solvents for a certain number of hours (see Figure 3), and, afterwards, the ORD curve was recorded. As expected, the most effective racemization medium was 1,2,4-TCBz, the solvent with the highest boiling point. In fact, heating in TCBz has caused the largest shift of the ORD curve toward the abscissa axis, i.e., the highest degree of racemization. However, it must be emphasized that these are just exploratory results not yet fine-tuned, neither in process nor in racemization yield.

### 2.4. Gigantic Optical Activity on PLLA Film

**Figure 4:**
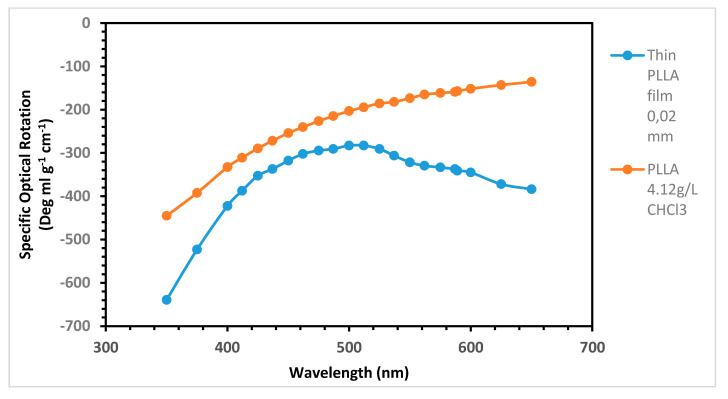
Optical rotatory dispersion (ORD) of a PLLA solution in CHCl_3_ (4.12 g/L) (orange line and dots) in comparison to a thin solid film of PLLA 20 μm thickness.

High molecular weight PLLA dissolved trichloromethane shows a negative plain ORD curve (Figure 4) in the spectral range between 350 and 650 nm of since the peak due to the Cotton effect associated with the n → π* transition of the ester chromophore of PLLA occurs at about 210–220 nm with a trough at 275 nm and a crossover at 240 nm [18]. Indeed, the PLLA absorption maximum due to the ester the n → π* electronic transition was measured at 209.8 nm on a 35 μm thin film [26,27]. Figure 4 shows also the ORD curve of a thin PLLA solid film. The thickness of the film was measured through the fringing effect method [27], while the specific optical rotation was calculated with a modification of Equation (1), as follows:

[α]_λ_ = α l^−1^ ρ^−1^, (2)

where the film thickness is expressed in dm as in Equation (1), and the concentration was substituted with the PLLA density (in g/mL), which is 1.290 [2]. The PLLA thin film curve is considerably shifted to more negative values of specific optical rotation with respect to the PLLA solution in CHCl_3_, and the shift appears much more pronounced between 500 and 650 nm, as well as between 350 and 400 nm. Considering the specific optical rotation at the D line of sodium (i.e., 589 nm), PLLA in CHCl_3_ shows [α]_589sol_ = −157, while PLLA as thin solid film shows the specific optical rotation [α]_589film_ = −341, a value more than double in the film than in solution. The highest specific optical rotation of PLLA in the ORD curve of Figure 4 can be observed at 350 nm with [α]_350sol_ = −445 in CHCl_3_, while, in the thin solid film, [α]_350film_ = −640, about 1.5 times larger in the solid state. These values are comparable to those reported by earlier investigators [42,43], and the higher rotation power observed in the solid state reflects the local environment of the polymer chains in supermolecular helical assemblies and, consequently, the solid state structure (morphology) of the polymers [42,43]. Indeed, more recent studies [17] have shown how the chiral bias transfers from molecular level (molecular chirality) to helical chain (conformational chirality) and then to helical superstructure or phase in the solid state (hierarchical chirality) and have developed chirooptical analytical techniques to distinguish the different contribution of each chirality level.

The giant optical activity exhibited by PLLA films can be further enhanced by uniaxially stretched films as demonstrated by some researchers [44,45], who have reported values as high as 7200°/mm when measured along the fiber axis, values about 300 times those measurable in α-quartz crystals. Further discussion about the solid state giant optical rotation power of PLLA crystals can be found in Reference [46].

### 2.5. Gigantic Optical Activity on PLLA Green Film Recovered from Used Water Bottles

**Figure 5:**
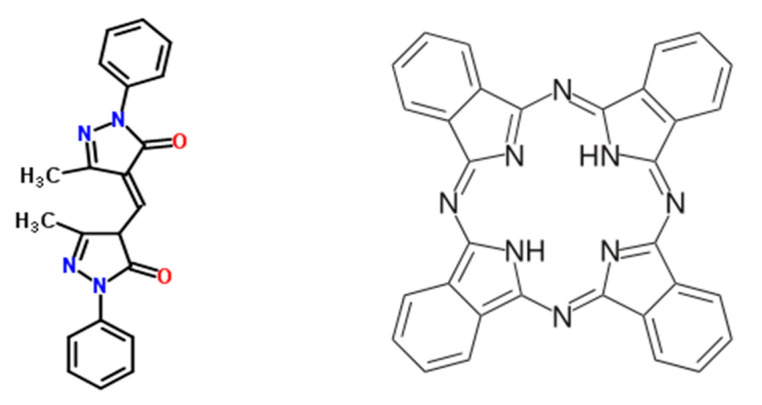
Chemical structure of Solvent Yellow 93 (SY93), which is a pyrazolone-dye (**left**) and phthalocyanine blue or Pigment Blue 16 (**right**).

The green coloration of PLLA or other plastics bottles (e.g., PET, polyethylene terephthalate) can be easily achieved by a combination of Solvent Yellow 93 (SY93) and very small amounts of phthalocyanine blue pigment like for instance Pigment Blue 16 (PB16) [47]. The latter pigment is a metal-free phthalocyanine (as shown in Figure 5), and it is characterized by a distinctly greener shade of blue than copper-phthalocyanine [48]. In Figure 5 are reported the chemical structures of these two colorants. SY93 is a pyrazolone-based dye characterized by two substituted pyrazolone structural units connected together by a conjugated methine bridge. The PB16 is a well-known and easily accessible pigment derived from phthalic anhydride [48].

**Figure 6:**
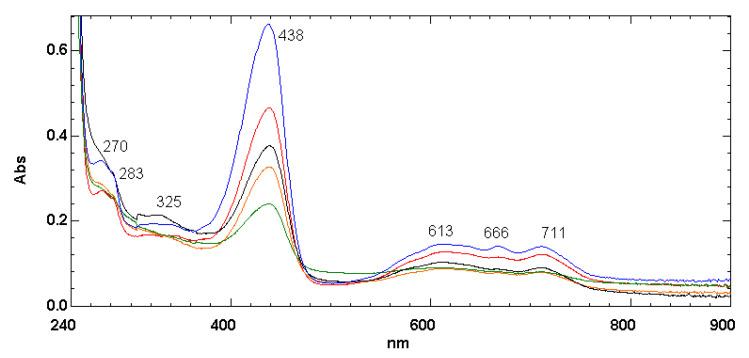
Electronic absorption spectra of PLLA green thin solid film (30 μm thick) from used water bottles. The blue line represents the absorption curve of the pristine film, while the red, black, orange, and green lines were collected, respectively, after 3, 6, 10, and 66 days of exposure to ozone.

Figure 6 (blue line) shows the electronic absorption spectrum of a pristine green thin PLLA film (30 μm thick) recovered from a used water bottle. PLLA is completely transparent in the visible and in the UV-A and UV-B spectral range. In fact, the main PLLA electronic transition occurs at 209.8 nm in the UV-C spectral window and is due to ketone band of the ester group [26,27]. Thus, in Figure 6, the main absorption band at 438 nm is due to the pyrazolone dye SY93 with a bright yellow color. On the other hand, the electronic absorption spectra of phthalocyanines are known in great detail [49,50,51]. The electronic transition at 325 nm in Figure 6 is the Soret, or B-band, which is always accompanied by the Q bands which, for PB16, appear at 613, 666, and 711 nm [49,50,51].

The exposure of the PLLA film containing SY93 and PB16 to ozone causes a gradual bleaching of the film, as shown in Figure 6. In fact, both the SY93 electronic band at 438 nm, as well as the B- and Q-bands of PB16, show a gradual reduction of intensity as function of the ozone exposure time.

**Figure 7:**
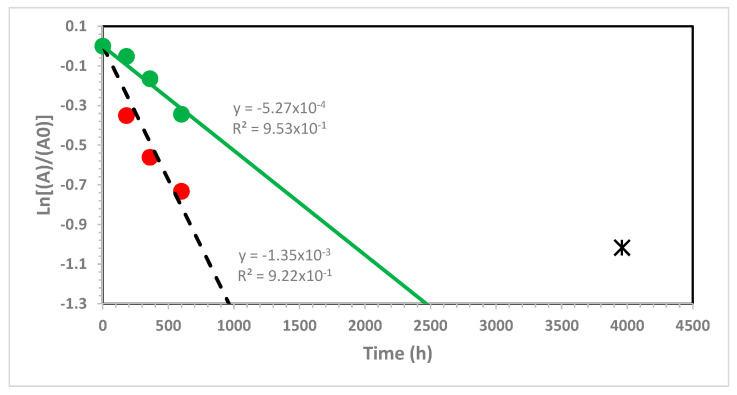
Kinetics of dyes bleaching inside the PLLA film (30 μm thick) exposed to ozone; ozonolysis of SY93 with k_dec_ = 1.35 × 10^−3^ h^−1^ (absorbance at 438 nm, red dots and black dotted line); the asterisk shows the value of Ln[(A/A_0_)_438_] after 4000 h exposure to ozone and not included in the determination of the SY93 ozonolysis rate constant; ozonolysis of PB16 with k_dec_ = 5.27 × 10^−4^ h^−1^ (absorbance at 711 nm, green dots and line).

Figure 7 shows the slow kinetics of the bleaching action of ozone on the thin PLLA film in which bright green color fades with time. Using the pseudofirst order kinetics law and applying it to the 428 nm absorbance, it is possible to determine the kinetics rate constant of SY93 decomposition, which is 1.35 × 10^−3^ h^−1^. The reaction of SY93 is easily explainable as ozone essentially attacks the methine bridge, breaking apart the two pyrazolone units, hence bleaching the yellow color. The phthalocyanine is also sensitive to ozone attack, and, when it is metal-free, it is even more prone to the aza-annulene ring breakdown [52]. However, due to some degree of aromatic character, the phthalocyanine is reacting with ozone with a relatively slower kinetics rate constant in comparison to other unsaturated compounds [52]. This is the case also of PB16. In fact, by treating the absorbance of the band at 711 nm according to the pseudofirst order kinetics, as shown in Figure 7, it is possible to obtain the kinetics rate constant for the phthalocyanine decomposition at 5.27 × 10^−4^ h^−1^, about one order of magnitude slower than the SY93 ozonoloysis rate constant.

The slow kinetics rate constants determined in Figure 7 suggest that the ozonolysis of SY93 and PB16 embedded in the PLLA thin film is diffusion controlled and depends on the ozone permeation and diffusion into the PLLA film and its migration inside the film to reach the two dyes. Thus, the degree of ozone exposure of the two dyes is identical, but SY93 reacts slightly faster than PB16 with ozone as determined. It is also interesting to notice that PLLA is completely not reactive with ozone. This is expected, since PLLA does not have ethylenic double bonds, but this was also checked experimentally by passing a stream of ozone through a CHCl_3_ solution of PLLA (concetration 0.4 g/100 mL). Neither changes in the viscosity of the solution were observed through viscosimetric measurements, nor development of any oxidation band in the infrared spectrum, even after prolonged ozone treatment.

**Figure 8:**
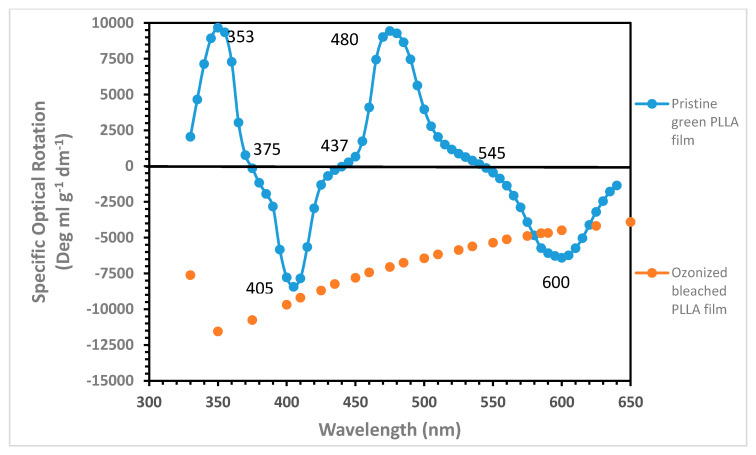
Optical rotatory dispersion (ORD) of thin solid film (30 μm thick) of PLLA (blue line and dots) from used water bottles with combined green and blue pigment with strong anomalous Cotton effect; same thin solid film of PLLA after exposure to ozone (orange line and dots); reference PLLA ORD curve 412 mg dissolved in 100 mL of CHCl_3_.

The most intriguing result of this paper is certainly the ORD measurement made on the green thin PLLA film from used water bottles reported in Figure 8 showing a multiple Cotton effect with a certain degree of complexity, while the ORD curve of a transparent PLLA thin film is just a plain negative curve, as shown previously in Figure 4. The multiple Cotton effect in the green PLLA film is due to an extrinsic Cotton effect derived from the interaction of the helical structure of PLLA in the solid state and the two dyes SY93 and PB16 [53], as well as the mutual interaction of the two dyes in a chiral environment represented by the PLLA matrix in the solid state [31]. Similar extrinsic Cotton effect, but with considerably smaller ORD amplitude and complexity, has been detected also in our earlier works dealing with PLLA-iodine and PLLA-C_60_ complexes [26,27]. Figure 8 shows also that the multiple Cotton effect observed in the ORD of the green PLLA film vanishes completely as soon as the two dyes SY93 and PB16 are decomposed into other not colored products after exposure of the PLLA film to ozone, demonstrating that the two dyes are the key source of the complex ORD spectrum of Figure 8. After the ozone treatment, the new ORD curve on PLLA film shows a plain negative trend with a through at about 350 nm and a peak beyond the spectral range accessible to our spectropolarimeter. Thus, a new Cotton effect in the ozonized PLLA film occurs below 300 nm and is due to the ozonolysis products of the dyes SY93 and PB16, which remain trapped in the bleached PLLA film.

For the interpretation of the complex ORD spectrum of PLLA green film with two dyes, we will refer to the excellent review work on chiroptical properties of thin films of π-conjugated systems made by Albano, Pescitelli, and di Bari [31].

The two dyes SY93 and PB16 represent two π-conjugated molecules having an approximately planar structure. In fact, SY93 is not fully planar since, in its chemical structure, one carbon atom and two nitrogen atoms are hybridized sp^3^. However, at least the pyrazolone chromoforic units are both π-conjugated and planar. In addition, the metal-free phthalocyanine is not fully planar because two nitrogen atoms are sp^3^ hybridized. This structure has only twofold axis of symmetry in contrast with the fourfold axis of symmetry observed in the metal-phthalocyanines [49,50]. The lower symmetry in the free-base phthalocyanine is manifested by higher spectral complexity since, in the electronic spectrum, the Q band of metal-substituted phthalocyanin is splitted in two doublets in the free base, the Q_y_ and the Q_x_ bands, often showing also vibrational fine structure [49,50]. This is the reason why we see at least four absorption bands in the spectra of Figure 6, with Q_y_ being the bands at 613 and 640 nm and Q_x_ the other doublet at 666 and 711 nm. Once SY93 and PB16 are embedded in chiral environment, they interact with each other and are stacking in a helical way, intercalating the isotactic PLLA left-helices arranged with the molecular residues on a nonintegral 10_3_ helix [19,20,44]. In other words, SY93 and PB16 give rise to a chiral stack guided by the PLLA helices and interact each other giving rise to exciton coupling due to mutual perturbation of the excited states. When several molecules are in close contact in a thin chiral film, exciton coupling occurs, which produces very a strong optical rotation signal, as the gigantic [α]_λ_ values we are observing in the ORD spectrum of Figure 8. The exciton coupling is easily recognized in chiroptical spectra because it gives rise to two bands of opposite sign and similar amplitude [31]. For instance the exciton couplet at 353 and 405 nm in Figure 8 is an example, followed by another one at 480 and 600 nm. The form of the bisignate exciton couplets in Figure 8 may suggest a preferential stacking of the dye molecules following left-handed helices, the same helical conformation observed on PLLA in the solid state, i.e., the chiral medium embedding the dyes.

The specific optical rotation reported in Figure 8 at any wavelength λ was calculated according to Equation (2) and, astonishingly, values as high as [α]_350_ = +9668 or [α]_480_ = +9436 and as low as as [α]_405_ = −8437 or [α]_D_ = −6080 and [α]_600_ = −6405 were obtained.

## 3. Materials and Methods

### 3.1. Materials and Equipment

*l*-(+)-lactic acid solution 88–92%, toluene and xylenes (mixture of isomers) were purchased from Aldrich-Merck (USA-Germany). Commercial PLLA for 3D printing was used as reference PLLA for ORD and other spectroscopies. It was characterized by a DSC (Differential Scanning Calorimetry) glass transition at +60 °C and by a DSC melting point peak at 157 °C with a melting enthalpy of 45.4 J/g. Green PLLA films were obtained from used plastic water bio-bottle from S. Anna company, Vinadio, Italy.

Spectrophotometric studies were performed on a Shimadzu UV-2450 spectrophotometer (made in Japan) equipped with thermostatted cells through the cell temperature controller TCC-240A. The optical rotatory dispersion (ORD) spectra were recorded on a Jasco P-2000 polarimeter (JASCO, Japan) equipped with a digital monochromator from Optometrics, model DMC1-03 from USA, which transforms the polarimeter into a spectropolarimeter. Infrared spectra were recorded on a Nicolet 6700 FT-IR spectrometer from ThermoFisher Scientific (Waltham, MA, USA) using reflectance mode and ZnSe crystal. The reflectance spectra were then converted into conventional infrared absorption spectra through the Omnic software of the spectrometer. In addition, the integration of the infrared absorption bands was made through the Omnic software of the spectrometer.

### 3.2. l-(+)-Lactic Acid Polycondensation Study to PLAO

*l*-(+)-lactic acid 90% solution (154,4 g) was heated with 200 mL of toluene in an esterification glassware apparatus equipped with a conventional Dean-Stark trap for the collection of the reaction water and the water entraining solvent under azeotropic distillation conditions. The mixture was stirred at 600 rpm and heated at 115 °C for about 12 h applying a discontinuous vacuum. At the end of this first polycondensation step, all toluene was recovered by distillation together with 29.5 mL of water. Since 26.2 mL were due to the natural water content of the pristine 90% lactic acid solution, only 3.3 mL were due to lactic acid polycondensation. The second step started with the addition of 150 mL of xylenes to PLAO and heating was started at 115 °C and maintained for 24 h, applying discontinuously for the first 12 h and continuously for the other 12 h. A total of 15.5 mL of reaction water was collected (including 3.3 mL of the first stage), together with the 150 mL of xylenes. Calculations show that, at the end of the second stage, 60% of the starting lactic acid has been oligomerized. The third stage was a repetition of the second stage, with the exception that the temperature was kept at 120 °C. At the end of the 3rd reaction stage, the total amount was 18.0 mL (including 3.3 mL of the 1st stage and 12.2 mL of the 2nd stage). The calculation shows that 70% of the starting lactic acid has been oligomerized. Small samples were taken from the PLAO at the end of each reaction stage for FT-IR and ORD measurements. Regarding the ORD measurement, the PLAO of 1st and 2nd reaction stage were dissolved in acetone (1.50 g/100 mL in both cases). PLAO of the 3rd stage was not soluble in acetone, and it was dissolved in CHCl_3_ (1.50 g/100 mL). High molecular weight reference PLLA was dissolved in CHCl_3_, as well (1.50 g/100 mL). The ORD measurement on *l*-(+)-lactic acid 90% was made directly in the neat solution, without any dilution. In all cases, 0.5 dm path length cell was used.

### 3.3. Partial Thermal Racemization of PLLA

High molecular weight commercial PLLA (202 g) was heated in 20.0 g of monochlorobenzene for 8 h to the refluxing temperature conditions (≈145 °C). After cooling, the solution was diluted with CHCl_3_ to a final volume of 50 mL in a volumetric flask and the ORD measured on this solution.

High molecular weight commercial PLLA (200 g) was heated in 20.0 g of ortho-dichlorobenzene (o-DCBz) for 4 h to the refluxing temperature conditions (≈180 °C). PLLA is not soluble in o-DCBz at room temperature, but it dissolves completely in hot o-DCBz and precipitates from the solution on cooling as white flakes. The resulting slurry was diluted with CHCl_3_ to a final volume of 50 mL in a volumetric flask, and the resulting homogeneous solution was submitted to ORD measurement.

High molecular weight commercial PLLA (200 g) was heated in 20.0 g of 1,2,4-trichlorobenzene (TCBz) for 28 h to 195 °C. PLLA is not soluble in TCBz at room temperature, but it dissolves completely in hot TCBz and precipitates from the solution on cooling as white flakes. The resulting slurry was diluted with CHCl_3_ to a final volume of 50 mL in a volumetric flask, and the resulting homogeneous solution was submitted to ORD measurement.

### 3.4. Preparation of PLLA Film

A solution of high molecular weight PLLA was prepared by dissolving 0.412 g PLLA in 100 mL of CHCl_3_. The solution was poured in a large Petri dish and left to evaporate slowly under a fume hood. A beautiful and uniform PLLA thin solid film was obtained and was removed from the dish with the aid of tweezers. The thickness of the free standing PLLA film was measured spectrophotmetrically by fringing effect method [27] and was found to be 20 μm thick. The film was then submitted to ORD measurement equipped with a specific sample holder designed for films.

### 3.5. Green PLLA Film from Used Water Bottle

A used water bottle made with green PLLA was cut into small sheets typically 1.0 cm × 3.0 cm, and the thickness of the sheets was measured with a digital micrometer from Somet. Only selected sheets with 30 μm thickness were used for the ORD measurements a using specific sample holder designed for films. Following the recommendations from Albano, Pescitelli, and Di Bari [31], the ORD measurements on the green PLLA films were recorded always on the front and the back of the film (i.e., flipping the film to the other face after the first measurement on the front face) and then averaging the resulting ORD curves. As explained in Reference [31], with this approach, it is possible to minimize some spurious signals and artifacts often occurring with ORD and CD measurements on thin films. A full discussion of this point is reported in Reference [31].

### 3.6. Exposure to Ozone and Kinetics of Ozone Bleaching of the Green PLLA Film from Used Water Bottle

A selected 30 μm thick PLLA green film sheet was used to record the electronic absorption spectrum and the ORD curve, as detailed in Section 3.5, and then it was transferred into a 2 L flask, which was evacuated and then filled with a mixture of O_3_/O_2_, with O_3_ being about 5% mol at the beginning of the exposure. The green color of the PLLA film was gradually bleached by the action of the ozone, which selectively causes the ozonolysis of the two dyes present in the PLLA film, namely SY93 and PB16 for which chemical structures are shown in Figure 5. Periodically, the PLLA film was removed from the flask, and the electronic spectrum recorded as shown in Figure 6. When the PLLA film was also completely bleached, the ORD curve was recorded as shown in Figure 8 and compared with the ORD curve of the pristine green PLLA film recorded before the ozone attack. The spectrophotometric absorption data were treated according to pseudofirst order kinetics law for the determination of the ozonolysis rate constants of the dyes SY93 and PB16 inside the PLLA matrix film.

## 4. Conclusions

It was found that the polycondensation of *l*-(+)-lactic acid to PLAO and then to PLLA can be followed by ORD spectroscopy or the related polarimetric measurement at 589 nm. The increase in molecular weight during the azeotropic polycondensation reaction leads to a continuous enhancement of the optical activity and a gradual shift of the ORD curves toward more negative [α]_λ_ values, as shown in Figure 1. This phenomenon is due to the ability of PLAO and PLLA to assume a helical conformation in solution. This superstructure is enhanced by the increase in molecular weight and reflected in the ORD measurements. Furthermore, it was shown in Figure 2 that also FT-IR spectroscopy is another effective tool useful to follow the oligomerization and then the polymerization of *l*-(+)-lactic acid.

The thermal racemization of PLLA was explored for the first time in high boiling chlorobenzenes (ClBz, o-DCBz, and TCBz) using ORD spectroscopy. Although the best reaction conditions were not optimized, it was found that the most effective solvent able to cause the PLLA partial racemization was TCBz as shown in Figure 3.

Giant optical activity was measured on PLLA thin solid films, as reported in Figure 4. This property may have applications in numerous different chirooptical devices involving display and wearable technologies, as well in efficient and large-area solar cells. Furthermore, PLLA recovered from used water bottles and loaded with two dyes SY93 and PB16 π-conjugated and approximately planar structure (see Figure 5) displays in the ORD spectrum an unusually beautiful multiple Cotton effect (Figure 8). Due to this unique effect derived from the interaction of PLLA chiral centers and helical structure with the mentioned dyes, the PLLA optical activity is further enhanced at certain wavelengths reaching really gigantic values, far larger than those measured on a PLLA film without dyes. Thin films with such unique chirooptical activities achieved by combining an optically active polymer with certain dyes have great potential applications in molecular electronics [31].

Green PLLA, recovered from used bottles, shows the just mentioned multiple Cotton effect and quite unique chirooptical properties. It has been found that the green PLLA film can be completely bleached by exposure to ozone (see kinetics curve in Figure 7). While the two dyes SY93 and PB16 are attacked and destroyed by ozone, the PLLA matrix was found fully resistant to ozone, and the resulting thin solid film retains its good mechanical properties, even after a prolonged exposure to ozone and a complete bleaching.

## Figures and Tables

**Table 1:** Integrated absorptivity of selected infrared bands of Lactic Acid, PLAO, and PLLA.

	Integr. abs.	Integr. abs.	Integr. abs.	Integr. abs.	Integr. abs.	Integr. abs.
	3450-20 cm^−1^	1750 cm^−1^	1129 cm^−1^	1456 cm^−1^	1224 cm^−1^	1096 cm^−1^
Lactic acid 88%	19.44	23.25	14.57	2.79	6.00	1.09
Lactic acid 100%	13.45	22.50	14.32	2.69	6.00	1.08
PLAO 1st stage	9.67	22.40	10.63	3.90	14.45	3.27
PLAO 2nd stage	4.40	22.20	6.07	4.07	15.68	6.92
PLAO 3rd stage	4.90	21.00	6.19	4.12	16.42	7.23
PLLA high Mw	2.03	14.40	3.99	7.50	20.00	7.30

## Data Availability

Data available in a publicly accessible repository that does not issue DOIs, e.g., Re-searchgate.
